# Sleep quality and anxiety among Egyptian population during covid-19 pandemic

**DOI:** 10.5935/1984-0063.20210020

**Published:** 2022

**Authors:** Samar Abd ElHafeez, Miguel Meira e Cruz, Salma Gouda, Marwa Nofal, Abdalrahman Fayed, Ramy Mohamed Ghazy, Jaidaa Mekky

**Affiliations:** 1 High Institute of Public Health, Alexandria University, Epidemioloy Department - Alexandria - Egypt.; 2 Centro Cardiovascular da Universidade de Lisboa, Lisbon School of Medicine, Sleep Unit - Lisbon - Portugal.; 3 Escola Bahiana de Medicina e Saúde Pública, Clinical Cronobiology and Sleep Medicine Group - Bahia - Brazil.; 4 Faculdade São Leopoldo Mandic, Neuroimune Pain Interface Lab - Campinas - São Paulo - Brazil.; 5 Centro Europeu do Sono, R&D Lab - Lisboa - Portugal.; 6 School of Medicine, Badr University - Cairo - Egypt.; 7 Helwan Mental Health Hospital, Sleep - Cairo - Egypt.; 8 High Institute of Public Health, Alexandria University, Health Administration and Behavioral Science Department - Alexandria - Egypt.; 9 High Institute of Public Health, Alexandria University, Tropical Health Department - Alexandria - Egypt.; 10 Faculty of Medicine, Alexandria University, Neuropsychiatry Department - Alexandria - Egypt.

**Keywords:** COVID-19, Sleep Quality, Anxiety, Egyptians

## Abstract

**Objectives:**

COVID-19 pandemic imposed a relevant number of stressful factors potentially impacting either daytime function or sleep quality. This study aimed to assess the sleep quality and anxiety among the general population living in Egypt.

**Material and Methods:**

A cross-sectional survey was conducted among 1,000 individuals who have been recruited via a convenience sample. A predesigned questionnaire was distributed online to collect data on sleep quality using the validated Arabic version of the Pittsburgh sleep quality index, anxiety disorders by the generalized anxiety disorder (GAD-7) scale, demographic characteristics, and clinical history.

**Results:**

Among the study participants (33.8% males, 46.2% healthcare workers, 25% had chronic diseases, 30.1% were physically active during lockdown, and 70.3% reported work termination due to COVID-19 infection, 68.4% complained of bad sleep and 70.3% showed clinically significant levels of anxiety). In multiple logistic regression analysis, bad sleep quality was significantly associated with moderate anxiety (OR: 1.88; [95% CI: 1.37-2.60]), severe anxiety (OR: 3.15; [95% CI: 2.18-4.55]), being physically active (OR: 0.53; [95% CI: 0.39-0.71]), received higher education as being postgraduate (OR: 0.56; [95% CI: 0.35-0.92]), or living with family (OR: 0.74; [95% CI :0.56-0.98]).The GAD-7 scale added 8% discrimination power for prediction of bad sleep quality compared to the model based on demographic and clinical data [with GAD: AUC=0.70, p<0.001; without GAD: AUC=0.62, p<0.001].

**Conclusion:**

COVID-19 had a significant impact on sleep quality and anxiety among Egyptians. Since both the conditions may overlap and potentiate each other leading to chronic dysfunctional outcomes, an accurate assessment and clinical approach may favor a better prognosis.

## INTRODUCTION

The new coronavirus pandemic is being considered the global health crisis of our time. The quick spread of coronavirus disease 2019 (COVID-19), which started in China on November 17^th^, 2019 led to its characterization as a pandemic by World Health Organization (WHO) on March 11^th^, 2020^1^. The pandemic has taken its toll on humanity especially the vulnerable and the least able to cope with its impacts^2^. Global efforts have been made to slow the spread of the virus by disease testing and treating cases, limiting travel, practicing social distancing, and cancelling or postponing different periodic and important events. These measures make the COVID-19 pandemic much more than a health crisis since it also negatively impacted social, economic, and political areas^3^.

On February 14^th^, 2020, Egypt announced its first COVID-19 confirmed case^1^. On March 16^th^, 2020 the country went on partial lockdown that lasted for about 90 days ending on June 27^th^, 2020. During the lockdown period, individuals had to deal not only with the uncertainty and insecurity about their health and their loved ones’, but they also had to experience major changes in their daily routine and lifestyle such as, for example, combining their work with homeschooling and household errands. These shifts on the common life patterns may have a significant impact on sleep duration and sleep quality. Furthermore, they were unable to get their usual rewarding time by engaging in activities or spending time with family and/or friends which in turn implies even more stress^4^. For those working from home, the disruption to established daily routines might lead to a deterioration of positive associations between home, relaxation, and sleep. Individuals may also have experienced lower levels of physical activity and higher caloric consumption in relation to stress. The combination of these factors may lead to sleep difficulties and other health risks^5^.

Several studies have addressed the psychological impact of the COVID-19 pandemic worldwide. The overall prevalence of anxiety symptoms, depressive symptoms, and poor sleep quality were 35.1%, 20.1%, and 18.2%, respectively, among the public affected by the COVID-19 outbreak in China^6^. In Taiwan, 55.8% of the participants reported sleep disturbance and 10.8% reported having suicidal thoughts^7^. Sleep problems were detected in 37.6% of the Greek population^8^ while 24.2% of Italians had moderate to extremely severe symptoms of depression, 32.6% had moderate to extremely severe symptoms of anxiety, and 50.1% complaint of moderate to extremely severe symptoms of stress^9^. The present study aimed to assess sleep quality and its determinants; and anxiety and its relationship with sleep among the general population living in Egypt during COVID-19 lockdown.

## MATERIAL AND METHODS

### Study design and population

A cross-sectional design was conducted using a non-random sampling technique (convenience sampling method) in order to recruit the population for this study. Data was collected using a predesigned structured questionnaire that was shared online to reduce face-to-face communication, according to the Egyptian regulations. EpiInfo version 7.2 was used to calculate the sample size. Based on the following criteria: population size of 10^8^, expected frequency of 18.2%^6^, a confidence level of 95%, and a margin of error of 5%; the minimal required sample size was 229. We amplified the sample size four times to compensate for any stratification and to overcome any invalid responses. People would be included in the study if they aged 18 years or older and were living in Egypt during the COVID-19 pandemic. The survey was shared through emails and social media platforms including Facebook, Twitter, and WhatsApp from April 25^th^ to June 1^st^, 2020. People completed the survey after reading the online informed consent and agreeing on participating in the study. There was no compensation (either financial or other) for participating in this study and it was not allowed to submit more than one survey. A total of 1,209 participants [Facebook (498), WhatsApp (457), and Twitter (254)] agreed to participate. Some of the participants were excluded: 123 had incomplete sheets, 66 aged <18 years old, 8 refused to participate after initial acceptance and 12 were living outside Egypt. The final sample was 1,000 adults living in Egypt.

### Data collection tools

The questionnaire was composed of three sections. The first section included questions on sociodemographic data (age, gender, education, residence, marital status, occupation, and work termination during COVID-19 lockdown), smoking history (non-smoker, ex-smoker, or current smoker intake of sleep medication, sleep problems before lockdown, following pandemic news, internet use (<2 hours, 2-4 hours, >4 hours), history of COVID-19 infection or knowing someone who had COVID-19 infection, history of dealing with COVID-19 cases, history of chronic diseases (diabetes mellitus, hypertension, cardiovascular diseases, and renal problems) or psychiatric illness, seeking for psychiatric consultation during lockdown, practicing physical activity, defined as anybody movement generated by the contraction of skeletal muscles that raises energy expenditure above resting metabolic rate, and is characterized by its modality, frequency, intensity, duration, and context of practice^10^, during lockdown (yes/no), weight and height, and living with family.

The second section derived from the validated Arabic version of the Pittsburgh sleep quality index (PSQI)^11^ to assess the sleep quality. The PSQI is a self-rated questionnaire that assesses seven components of sleep quality during the previous month: subjective sleep quality, sleep latency, sleep duration, habitual sleep efficiency, sleep disturbances, use of sleeping medication, and daytime dysfunction. A total of 19 items were rated on a scale scored from 0-3 (0, not during the past month; 3, ≥3 times a week), with the total PSQI score ranging from 0 to 21 with 0 indicating no sleep issues and 21 severe sleep difficulties and low sleep quality. A global score of >5 indicating poor sleep quality^12^.

The third section was composed of the Arabic version of the seven-item scale the generalized anxiety disorder scale (GAD-7) to measure anxiety level^13^. Participants were asked how often they were bothered by each symptom during the past two weeks. The response options were “not at all”, “several days”, “more than half the days” and “nearly every day”, and scored as 0, 1, 2, and 3, respectively. The scores for symptom severity were 5-9 for mild, 10-14 for moderate, and 15-21 for severe^14^.

### Ethical consideration

The study protocol was approved by the ethics committee of the Faculty of Medicine, Alexandria University in accordance with the international ethical guidelines for epidemiological studies^15^.

### Statistical analysis

The results are presented as mean and standard deviation (SD) in case of normally distributed data, median, and interquartile range (IQR) for non‐normally distributed data, or as a percentage for categorical data. The total score of the seven components of PSQI was calculated. The component score for the each component of PSQI was computed as follows; subjective sleep quality (component 1) was composed of the sub-score for item 9, sleep latency (component 2) was consisted of the added sub-score for items 2 and 5a, sleep duration (component 3) included the added sub-score for item 4, sleep efficiency (component 4) was assessed by adding the sub-score for items 1, 3, and 4, sleep disturbance (component 5) was calculated by summing the sub-score for items 5b to 5j, use of sleep medication (component 6) was composed of the sub-score for item 6, and daytime dysfunction (component 7) was composed of the added sub-score for items 7, and 8. Comparison of the total scores and component score by the sleep status (good versus bad) was done using t-test while chi-square was used to compare the responses for each item by the sleep status (good versus bad). Cross-tabulation of categorical data by sleep quality (good versus bad) with testing the association by chi-square test and McNemar’s test are also presented. In order to compare continuous variables t-test or Mann-Whitney test were applied. Pearson’s correlation analysis was used to test the relation between the PSQI scale and GAD-7 scale. The association between the different levels of anxiety (mild, moderate, and severe) and sleep status (good and bad) was tested by chi-square.

To identify the independent predictors of bad sleep quality among our study population, we built multiple logistic regression model. The variables will be included in the model if they have *p*<0.15 in the univariate analysis^16^. Interaction between all the independent predictors of sleep quality was investigated by calculating the product of each predictor with each other and by introducing this multiplication term into the multiple logistic regression model already including the two factors of the product as separate variables^17^. The final model included the following variables: age group, education level, history of living with family, follow-up of the pandemic news, history of psychiatric illness, history of psychiatric visit during the pandemic, history of sleep problems before the pandemic, history of physical activity during pandemic, and anxiety level. The odds ratio (OR) and 95% confidence interval (CI) were reported for all variables.

In order to identify the predictive power of anxiety for sleep quality beyond and above what was provided by the standard determinants, we constructed two logistic regression models. The first model included age group, education level, history of living with family, follow-up of the pandemic news, history of psychiatric illness, history of psychiatric visit during the pandemic, history of sleep problems before the pandemic, history of physical activity during pandemic, and GAD-7 scale and the second model included the same variables without GAD-7 scale. Further, the discriminatory power of GAD-7 scale in predicting bad sleep quality was calculated through performing receiver operating characteristics (ROC) analysis^18^. Data analyses were performed using the SPSS software (version 25 for Windows, SPSS Inc., Chicago, IL, U.S.).

## RESULTS

### Participant characteristics

Table 1 summarizes the baseline characteristics of the study population. Almost 66% aged between 18-34 years, 33.8% were males, 88.6% lived in urban areas, 43.3% had postgraduate degree, 10.3% were smokers, 49.9% single, 56.2% were living with family, 46.2% were healthcare workers, 70.3% reported to stop their work due to COVID-19 infection, 25% had chronic diseases, 30.1% were physically active during the lockdown, and 54.3% had normal body mass index. More than half (54.1%) of the study population slept less during the lockdown period, 39.6% had sleep problems before pandemic, 56.2% were on sleep medications, 73.2% followed the pandemic news, 62.3% spent more than 4 hours/day on the internet, 2.5% had COVID-19 infection, and 45.5% knew someone who had COVID-19 positive diagnosis.

**Table 1:** Baseline characteristics of the general population living in Egypt during COVID-19 pandemic.

Variables	Total sample N=1,000(%)	Good sleep quality n=316(%)	Bad sleep quality n=684(%)	*p*-value
**Age groups** 18-34 35-44 >45	656 (65.6) 256 (25.6) 88 (8.8)	194 (61.4) 94 (29.7) 28 (8.9)	462 (67.5) 162 (23.7) 60 (8.8)	0.11
**Gender** Male Female	338 (33.8) 662 (66.2)	117 (37) 199 (63)	221 (32.3) 463 (67.7)	0.15
**Residence** Urban Rural	886 (88.6) 114 (11.4)	282 (89.2) 34 (10.8)	604 (88.3) 80 (11.7)	0.67
**Education** Less than university Graduate Postgraduate	133 (13.3) 434 (43.4) 433 (43.3)	30 (9.5) 133 (42.1) 153 (48.4)	103(15.1) 301(44.1) 279(40.8)	0.02
**Smoking history** Non/Ex- smokers current smokers	897 (89.7) 103 (10.3)	280 (88.6) 36 (11.4)	617 (90.2) 67 (9.8)	0.44
**Marital status** Single Married Divorced Widowed	494 (49.9) 456 (45.6) 45 (4.5) 5 (0.5)	142 (44.9) 159 (50.3) 13 (4.1) 2 (0.6)	352 (51.5) 297 (43.4) 32 (4.7) 3 (0.4)	0.21
**Living with family**	561 (56.2)	190 (60.3)	371 (54.2)	0.07
**Occupation** Not healthcare workers Healthcare workers	538 (53.8) 462 (46.2)	179 (56.6) 137 (43.4)	359 (52.5) 325 (47.5)	0.22
**Work termination due to lockdown**	733 (73.3)	237 (75)	496(72.5)	0.41
**Body mass index** Underweight Normal weight Overweight Obese	177 (18.0) 535 (54.3) 207 (21.0) 67 (6.8)	55 (17.6) 172 (55.0) 63 (20.1) 23 (7.3)	122 (18.1) 363 (53.9) 144 (21.4) 44 (6.5)	0.93
**History of chronic diseases**	250 (25.0)	72 (22.8)	178 (26.0)	0.27
**Physical activity practice during lockdown**	301 (30.1)	124 (39.2)	177 (25.9)	<0.001
**Sleep less during lockdown**	541 (54.1)	166 (52.5)	375 (54.8)	0.49
**Sleep problems before pandemic**	396 (39.6)	135 (42.7)	261 (38.2)	<0.001*
**Sleep medications**	872 (87.2)	282 (89.2)	590 (86.3)	0.19
**Number of coffee cups per day** 0-1 2-5 >5	650 (65) 339 (33.9) 11 (1.1)	201 (63.6) 110 (34.8) 5 (1.6)	449 (65.6) 229 (33.5) 6 (0.9)	0.54
**Follow pandemic news**	723 (73.2)	240 (75.9)	483 (70.6)	0.08
**Internet hours** <2 2-4 >4	71 (7.1) 306 (30.6) 623 (62.3)	26 (8.2) 101 (32.0) 189 (59.8)	45 (6.6) 205 (30.0) 434 (63.5)	0.46
**History of COVID-19 infection**	25 (2.5)	6 (1.9)	19 (2.8)	0.41
**Knowing someone having COVID-19 infection**	445 (45.5)	132 (41.8)	313 (45.8)	0.24
**Dealing with COVID-19 patients**	124 (12.4)	39 (12.3)	85 (12.4)	0.97
**History of any psychiatric illness**	238 (23.8)	65 (20.6)	173 (25.3)	0.10
**History of psychiatrist visit during lockdown**	104 (10.4)	24 (7.6)	80 (11.7)	0.04
**GAD scale** Mean ±SD Median (IQR)	8.05±5.21 7 (4-12)	6.39±4.85 5 (3-8)	8.82±5.18 8 (5-13)	<0.001
**Body mass index** Underweight Normal weight Overweight Obese	177 (18.0) 535 (54.3) 207 (21.0) 67 (6.8)	55 (17.6) 172 (55.0) 63 (20.1) 23 (7.3)	122 (18.1) 363 (53.9) 144 (21.4) 44 (6.5)	0.93

### Sleep quality

More than two-thirds (68.4%) of the study participants complained about bad sleep. Participants with bad sleep quality were less educated (15.1% vs. 9.5%), less physically active (39.2% vs. 25.9%), gave a history of psychiatric visit during the pandemic (11.7% vs. 7.6%), and more anxious compared to participants with good sleep quality (Table 1). The seven subscales of PSQI are shown in Table 2. More than half of the study population (51.9) reported fairly good sleep quality, 42.6% took less than 15 minutes to fall asleep during the past month, 41.1% sleep for 6 to 7 hours, 54.9% had good sleep efficiency, 59.3% complaint of sleep disturbance less than once a week, 70.6% did not have trouble to stay awake during the past month, and 44.2% had somewhat problems to keep up enough enthusiasm to get things done. The mean PSQI total scores (3.74±1.13 vs. 9.61±3.03) and component scores of the whole sample; subjective sleep quality (0.70±0.57 vs. 1.72±0.88), sleep latency (1.62±0.64 vs. 2.00±0.89), sleep duration (0.63±0.49 vs. 1.12±0.97), sleep efficiency (0.90±0.31 vs. 1.22±0.18), sleep disturbance (1.00±0.43 vs. 1.54±0.58), use of sleep medication (0.40±0.22 vs. 0.95±0.39), daytime dysfunction (0.96±0.63 vs. 1.58±0.73) were significantly different between good and bad sleep quality groups (*p*<0.001).

**Table 2:** PSQI total and component scores among the general population living in Egypt during COVID-19 pandemic.

PSQI components	Total sample (N=1000)	Good sleep quality	Bad sleep quality	Significance
**PSQI total score**	7.78±3.74	3.74±1.13	9.61±3.03	
**Component 1: subjective sleep quality** Very good Fairly good Fairly bad Very bad	128 (12.8) 519 (51.9) 177 (17.7) 175 (17.5)	109 (34.8) 191 (60.9) 12 (3.7) 2 (0.6)	19 (2.8) 328 (47.9) 165 (24.1) 173 (25.2)	*p*<0.001
**Component 1 (mean ±SD)**				
Component 2: sleep latency	1.74±0.92	0.70± 0.57	1.72± 0.88	*p*<0.001
**During the past month, how long (in minutes) has it usually taken you to fall asleep each night?** <15 minutes 16-30 minutes 31-60 minutes >60 minutes	426 (42.6) 212 (21.2) 228 (22.8) 134 (13.4)	248 (78.9) 52 (16.1) 14 (4.3) 2 (0.6)	178 (26.1) 160 (23.5) 214 (31.2) 132 (19.3)	*p*<0.001
**Cannot get to sleep within 30 minutes** Not during past month Less than once a week Once or twice a week Three or more times a week	220 (22) 220 (22) 282 (22.8) 278 (27.8)	164 (52.2) 106 (33.5) 42 (13) 4 (1.2)	56 (8.2) 114 (16.7) 240 (35.1) 274 (39.9)	*p*<0.001
**Component 2 (mean±SD)**	1.57±0.43	1.62±0.64	2.00±0.89	*p*<0.001
**Component 3: Sleep duration** > 7 hours 6-7 hours 5-6 hours < 5 hours	399 (39.9) 411 (41.1) 101 (10.1) 89 (8.9)	203 (64.6) 111(34.8) 2 (0.6) 0 (0)	196 (28.6) 300 (43.9) 99 (14.4) 89 (13)	*p*<0.001
Component 3 (mean±SD)	1.90±0.43	0.63±0.49	1.12±0.97	*p*<0.001
**Component 4: sleep efficiency** > 85% 75-84% 65-74% < 65%	549 (54.9) 189 (18.9) 111 (11.1) 152 (15.2)	289 (91.3) 26 (8.1) 2 (0.6) 0 (0)	260 (38.2) 163 (23.8) 109 (15.9) 151 (22.1)	*p*<0.001
**Component 4 (mean±SD)**	1.87±0.11	0.90±0.31	1.22±0.18	*p*<0.001
**Component 5: sleep disturbance** Not during past month Less than once a week Once or twice a week Three or more times a week	31 (3.1) 593 (59.3) 346 (34.6) 29 (2.9)	29 (9.3) 257 (81.4) 30 (9.3) 0(0)	2 (0.3) 336 (49.3) 316 (46) 29 (4.2)	*p*<0.001
**Component 5 (mean ±SD)**	1.56±0.59	1.00±0.43	1.54±0.58	*p*<0.001
**Component 6: use of sleep medication** Not during past month Less than once a week Once or twice a week Three or more times a week	844 (84.4) 68 (6.8) 23 (2.3) 64 (6.4)	306 (96.9) 8 (2.5) 2 (0.6) 0 (0)	539 (78.8) 60 (8.8) 21 (3.1) 64 (9.3)	*p*<0.001
**Component 6 (mean ±SD)**				
Component 7: daytime dysfunction	1.02±0.35	0.40±0.22	0.95±0.39	*p*<0.001
**During the past month, how often have you had trouble staying awake while driving, eating meals,** **or engaging in social activity?** Not during past month Less than once a week Once or twice a week Three or more times a week	706 (70.6) 148 (14.8) 103 (10.3) 43 (4.3)	280 (88.3) 22 (6.8) 12 (3.7) 2 (0.6)	426 (62.3) 126 (18.4) 91 (13.3) 41 (5.9)	*p*<0.001
**During the past month, how much of a problem has it been for you to keep up enough enthusiasm to get things done?** No problem at all Only a very slight problem Somewhat of a problem A very big problem	95 (9.5) 218 (21.8) 442 (44.2) 245 (24.5)	65 (20.5) 92 (29.2) 131 (41.6) 28 (8.7)	30 (4.5) 126 (18.4) 311 (45.3) 217 (31.7)	*p*<0.001
**Component 7 (mean±SD)**	1.93±0.75	0.96±0.63	1.58± 0.73	*p*<0.001

Note:

* PSQI: Pittsburgh sleep quality index.

### Anxiety and its association with sleep quality

**Figure 1:**
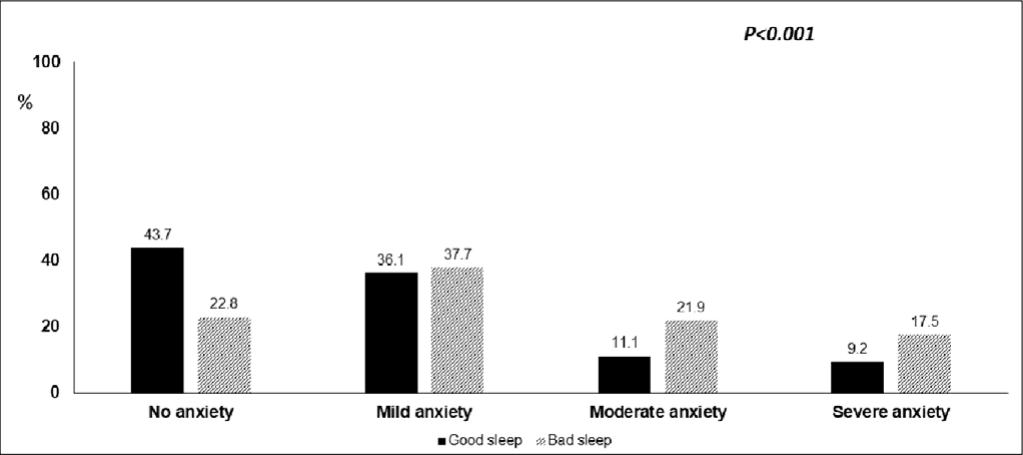
Relationship between anxiety levels and sleep quality among general population living in Egypt during COVID-19 pandemic.

**Figure 2:**
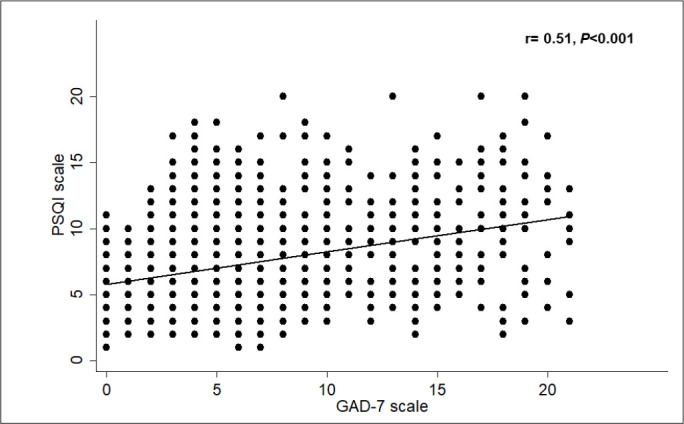
Correlation between GAD-7 scale for anxiety and PSQI for sleep quality.

Table 3 and Figure 1 presents the different anxiety levels among the study participants. There were 70.6% had some degree of anxiety. Based on anxiety level, 18.5% and 14.9% complaint of moderate and severe anxiety, respectively. Different anxiety levels were significantly higher among participants with bad sleep quality (*p<0.001*). The correlation between GAD-7 scale and PSQI scale was significant (r= 0.51, *p<0.001*) (Figure 2).

**Table 3:** Anxiety level among general population living in Egypt during COVID-19 pandemic.

Anxiety level	Total sample N=1,000 (%)	Good sleep quality n=316(%)	Bad sleep quality n=684(%)
**No anxiety**	294 (29.4)	138 (43.7)	156 (22.8)
**Mild**	372 (37.2)	114 (36.1)	258 (37.7)
**Moderate**	185 (18.5)	35 (11.1)	150 (21.9)
**Severe**	149 (14.9)	29 (9.2)	120 (17.5)

### Predictors of sleep quality among the study population

Table 4 showed that anxiety level increased the risk of bad sleep quality. Participants with moderate anxiety had two times odds of having bad sleep quality (OR: 1.88; [95% CI: 1.37-2.60]), while those with severe anxiety had three times odds complaining of bad sleep quality (OR: 3.15; [95% CI: 2.18-4.55]). On contrary, being physically active (OR: 0.53; [95% CI: 0.39-0.71]), received higher education as being postgraduate (OR: 0.56; [95% CI: 0.35-0.92]), or living with family (OR: 0.74; [95% CI :0.56-0.98]) reduced the odds of bad sleep quality.

**Table 4:** Predictors of bad sleep quality among general population living in Egypt during COVID-19 pandemic.

Variables	Unit of increase	OR (95% CI)	p-value
**Age groups**	1=> 45 years, 0=< 45 years	0.84 (0.62-1.13)	0.24
**Living with family**	1=yes, 0=no	0.74 (0.56-0.98)	0.04
**Graduate**	1=graduate, 0=others	0.67 (0.41-1.08)	0.10
**Postgraduate**	1=postgraduate, 0=others	0.56 (0.35-0.92)	0.02
**Practicing physical activity during lockdown**	1=yes, 0=no	0.53 (0.39-0.71)	<0.001
**History of sleep problems before the pandemic**	1=yes, 0=no	0.84(0.63-1.11)	0.23
**Follow pandemic news**	1=yes, 0=no	0.75 (0.55-1.04)	0.10
**Psychiatric visit during pandemic**	1=yes, 0=no	1.41 (0.83-2.39)	0.19
**psychiatric illness before pandemic**	1=yes, 0=no	1.21 (0.84-1.75)	0.30
**moderate anxiety**	1=yes, 0=no	1.88 (1.37-2.60)	<0.001
**Severe anxiety**	1=yes, 0=no	3.15 (2.18-4.55)	<0.001
**Constant**		3.89	0.02

### Discriminatory power of GAD scale in predicting participants with bad sleep quality

**Figure 3:**
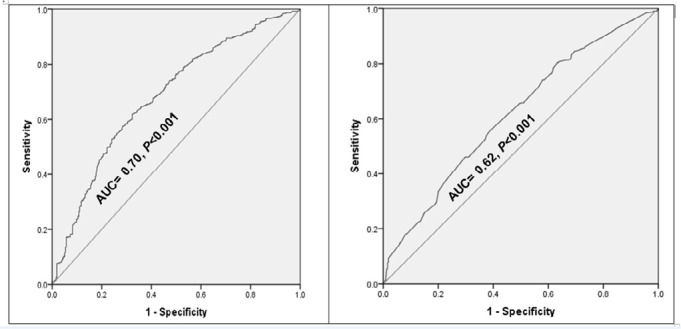
ROC curve analysis for discriminatory power of GAD scale in predicting sleep quality among general population living in Egypt during COVID-19 pandemic.

The area under the curve (AUC) was estimated for ROC curves from both models with GAD scale and model without GAD scale (Figure 3A and 3B, respectively). Generalized anxiety disorder scale added 8% discrimination power for the prediction of bad sleep quality compared to the model based on age group, education level, history of living with family, follow-up of the pandemic news, history of psychiatric illness, history of psychiatric visit during the pandemic, history of physical activity during pandemic [with GAD: AUC=0.70, *p*<0.001; without GAD: AUC=0.62, *p*<0.001].

## DISCUSSION

The present study assessed sleep quality and anxiety among the general population in Egypt. We found that 68.4% complaint of bad sleep quality and 70.6% experienced some forms of anxiety during the COVID-19 pandemic. We also identified anxiety as the main risk factor, while physical activity, receiving higher education, and living with family were protective factors for bad sleep.

Our findings are in line with the available data from previous literature. Several studies showed that sleep disorders prevail among 30-80% of the different groups of population^19-25^. In Arab countries, there was a high prevalence of sleep disorder, especially insomnia (56.0%) and daytime sleepiness (9.9%) among the Moroccan population^26^, 76% of the Jordanian university students were bad sleepers during the pandemic in the last spring^27^, while 23.1% of Egyptians complaint of inadequate sleep^28^. All components of PSQI were significantly different between participants with good versus bad sleep quality. Previous studies showed similar findings that PSQI components were lower among people identified as bad sleepers during COVID-19 pandemic^6,9,29,30^. Among the 7 subscales of PSQI, more than half of the Egyptian population reported fairly good subjective sleep quality, good sleep efficiency, and sleep disturbance for less than once a week. Hinz et al. (2017)^31^ demonstrated that among the German population 62.4% had fairly good subjective sleep quality, 31.7% needed less than 15 minutes to fall asleep, 26.5% sleep for 6 to 7 hours, and only 3.3% use sleep medication for less than once a week. Differences in the socioeconomic levels and the drug availability may clarify the differences between this study and our current results, especially in the use of sleep medications. The changes in sleep latency could be a result of excessive screen time at night. Decreased physical activity with subsequent little sun exposure as a consequence of home lockdown lead to changes in circadian rhythm maintenance^32,33^. Subsequently, sleep behaviour would be aggravated, and mood would be disturbed^34^. This in turn will reduce immunity and disturb body metabolism and energy^35^.

The emergence of COVID-19, with its rapid spread, has exacerbated anxiety in populations globally. Recently published systematic reviews showed that anxiety was prevalent among 20 to 50% of the population during the COVID-19 pandemic^36-38^. In Egypt, studies showed that anxiety was prevalent among almost 50-75% of the Egyptian population during the COVID-19 pandemic^28,39^. Before the pandemic, 4.75% of the Egyptians had some forms of anxiety according to the national survey of the prevalence of mental disorders in Egypt. This reflects the enormous impact of the pandemic among the Egyptian population^40^.

The variation in the prevalence of anxiety among the general population could be explained by the different geographical and sociodemographic criteria of the enrolled population, the different tools used to assess the psychological problems, and the availability of robust psychosocial and mental health support.

The pandemic imposed severe changes in the lifestyle of different populations with a major effect on sleep. These lifestyle changes (e.g., being forced to stay at home, work from home, or extended working hours with worrying about the possible health risks especially with an increased number of patients, all of this can have a considerable impact on daily functioning and night-time sleep^5^. The prolongation of such conditions leads to chronic stress and increased arousal leading to sleep-wake alterations. One part of this arousal response is anxiety. People respond to life stressful conditions by promoting arousal through the corticotropin-releasing hormone system and the locus ceruleus-autonomic nervous system. Activation of these two systems results in the release of norepinephrine and corticotrophin releasing hormone^41^. Anxious patients show difficulties in initiating and maintaining sleep and had increased time awake. They also complain of poor quality of sleep characterized by initial or middle insomnia and restless broken sleep. This could be explained by CNS hyper-vigilance and hyperarousal, as actual symptoms of anxiety, lead to nocturnal insomnia^42^.

Findings from our survey indicated that physical activity, high education, or living with families were conducive to reduce sleep disturbance. Physical activity creates arousal and triggers the release of endorphins, noradrenaline, serotonin, and dopamine, which can cause “exercise-induced euphoria”, which promotes a number of positive feelings such as peacefulness, safety, and confidence. This helps in improving mood and sleep quality^43,44^. During the lockdown in Egypt, all fitness centers and public places were shut down, which made it difficult especially for adolescents to engage in physical activities^4^. About 30% of the residents in our study sample reported that they were able to practice physical activity during lockdown. Appropriate alternatives to go outside for exercise, like following online exercise videos, taking virtual classes, exercising with families, or tackling calorie-burning chores should be promoted among population. This will help in improving sleep quality and reactions to stress from the epidemic.

Education level impacts sleep quality through increasing the awareness and compliance with the prevention and control measures of the COVID-19 epidemic^19^. Therefore, during isolation or quarantine, residents with higher education levels may have adopted more proactive coping patterns, such as reading, physical activity, and seeking psychological support from family.

Living with families was another favorable factor against complaining of poor sleep. Previous studies have indicated that death of parents in childhood, not living with parents, and parents’ psychological problems and mental illnesses provoke emotional and anxiety disorders in adults^45,46^. People who are deprived of emotional support either from family or society showed worse psychological consequences compared with their counterparts who were offered social care^28^.

The strength of our study lies in being comprehensively investigating sleep quality among the general population in Arab countries during the COVID-19 pandemic, using a specific validated sleep questionnaire (PSQI). The Egyptian study reported the sleeping hours per day using depression anxiety stress scale-21^28^. While the Moroccan study aimed to assess drowsiness level among participants by Epworth sleepiness scale^26^. Our study has limitations. First, it was conducted as a web-based survey that may introduce selection or no-response bias. However, it was completely effective for the research objectives, because it facilitated the wide dissemination of the survey questionnaire during a period where, due to the pandemic, there are many territorial restrictions. The latest data reported by the annual Egyptian report on the use of the internet shows that Internet penetration stood at 54% in January 2020 and the number of mobile connections in Egypt was equivalent to 91% of the total population^47^. This technique ensured the safety of both interviewers and interviewees. Second, we used a subjective question (yes/no) to ask about the history of sleep problems before the pandemic although, using a validated tool to assess sleep disturbance would have been more informative. We compared our findings to that reported from previous study, which has been done before the COVID-19 pandemic among Egyptians, using the PSQI scale. It showed that 36% of them suffered from sleep disorders^48^. This implies the drastic effect of lockdown on sleep pattern among Egyptians. We were not able to assess the history of anxiety before the pandemic. However, data from a national previous survey showed that anxiety was not so prevalent among the Egyptian population compared to what has been reported from our findings^40^. Third, we used non-random sampling technique (convenience sampling method), however, this method was the most appropriate due to national lockdown and poor access to the community members. Finally, this study remains an observational study with limited ability to assess causality, control for unmeasured confounders or evaluate the stability of the responses.

Our study has important implications on health policy and clinical practice. Population with sleep problems and anxiety is one of the vulnerable groups that should be given attention and support for their well-being during the pandemic. This support may be in the form of psychological aids and psycho-educational interventions on sleep and circadian rhythms to maintain a normal sleep-wake schedule and daily routine during periods of isolation^49^. In addition, it is crucial to raise awareness about the psychosocial implications of the pandemic including its effect on sleep quality among the public and health care providers and offer early diagnosis and management. Egyptian Ministry of Health and Population allocated two hotlines for psychological support during the COVID-19 crisis. Our data also have implications for future research. Considering anxiety is a main determinant for bad sleep quality, studies should focus on investigating the relationship between anxiety and sleep quality in a prospective manner. Finally, long-term follow-up and outcomes of lockdown on the psychological health of population need to be included in future studies to examine the post-pandemic impact.

## CONCLUSION

In conclusion, sleep quality is poor among people from Egypt during the COVID-19 pandemic. Anxiety is the main risk factor for poor sleep quality. Physical activity, living with family, and receiving higher education help to improve sleep pattern. Further studies are needed to estimate the long-term effect and prognosis of COVID-19 on the psychological health of the Egyptian population.
